# Socio-economic and demographic determinants of non-communicable diseases in Kenya: a secondary analysis of the Kenya stepwise survey

**DOI:** 10.11604/pamj.2020.37.351.21167

**Published:** 2020-12-16

**Authors:** Kibachio Joseph Mwangi, Valerian Mwenda, Gladwell Gathecha, David Beran, Idris Guessous, Oren Ombiro, Zachary Ndegwa, Peninnah Masibo

**Affiliations:** 1 Faculté de Médecine, Université de Genève, Genève, Suisse,; 2 Division of Non-communicable Disease, Ministry of Health, Nairobi, Kenya,; 3 Field Epidemiology and Laboratory Training Program, Ministry of Health, Nairobi, Kenya,; 4 Division of Primary Care Medicine, Department of Primary Care Medicine, Geneva University Hospitals, Geneva, Switzerland,; 5 Division of Tropical and Humanitarian Medicines, Geneva University Hospitals and University of Geneva, Geneva, Switzerland,; 6 Improving Public Health Management for Action (IMPACT) Program, Ministry of Health, Nairobi, Kenya,; 7 Global Programs for Research and Training, University of California, San Francisco, Nairobi, Kenya

**Keywords:** Non-communicable diseases, socio-demographic, risk factors, determinants, Kenya

## Abstract

**Introduction:**

non-communicable diseases (NCDs) are projected to become the leading cause of death in Africa by 2030. Gender and socio-economic differences influence the prevalence of NCDs and their risk factors.

**Methods:**

we performed a secondary analysis of the STEPS 2015 data to determine prevalence and correlation between diabetes, hypertension, harmful alcohol use, smoking, obesity and injuries across age, gender, residence and socio-economic strata.

**Results:**

tobacco use prevalence was 13.5% (males 19.9%, females 0.9%, p<0.001); harmful alcohol use was 12.6% (males 18.1%, females 2.2%, p<0.001); central obesity was 27.9% (females 49.5%, males 32.9%, p=0.017); type 2 diabetes prevalence 3.1% (males 2.0%, females 2.8%, p=0.048); elevated blood pressure prevalence was 23.8% (males 25.1%, females 22.6%, p<0.001), non-use of helmets 72.8% (males 89.5%, females 56.0%, p=0.031) and seat belts non-use 67.9% (males 79.8%, females 56.0%, p=0.027). Respondents with <12 years of formal education had higher prevalence of non-use of helmets (81.7% versus 54.1%, p=0.03) and seat belts (73.0% versus 53.9%, p=0.039). Respondents in the highest wealth quintile had higher prevalence of type II diabetes compared with those in the lowest (5.2% versus 1.6%,p=0.008). Rural dwellers had 35% less odds of tobacco use (aOR 0.65, 95% CI 0.49, 0.86) compared with urban dwellers, those with ≥12 years of formal education had 89% less odds of tobacco use (aOR 0.11, 95% CI 0.07, 0.17) compared with <12 years, and those belonging to the wealthiest quintile had 64% higher odds of unhealthy diets (aOR 1.64, 95% CI 1.26, 2.14). Only 44% of respondents with type II diabetes and 16% with hypertension were aware of their diagnosis.

**Conclusion:**

prevalence of NCD risk factors is high in Kenya and varies across socio-demographic attributes. Socio-demographic considerations should form part of multi-sectoral, integrated approach to reduce the NCD burden in Kenya.

## Introduction

Non-communicable diseases (NCDs) are currently the leading cause of both morbidity and mortality worldwide, with indications that this trend will continue increasing [1]. Low- and middle-income countries (LMIC) contribute to more than 80% of global premature NCD deaths [2]. Almost 30 percent of NCD deaths in LMICs occur among people under age 60 years at the peak of their economic productivity compared to only 13 percent in high-income countries [3,4]. This has a major impact on economic livelihoods of individuals, their families and health systems due to their long, chronic courses, need for lifelong treatment and follow-up and need for advanced methods for management of complications [5]. With the exception of the African region, globally NCD mortality exceeds that of communicable, maternal, perinatal and nutritional conditions combined [6]. By 2030, NCDs are estimated to contribute to three times as many disability-adjusted life years (DALYs) and nearly five times as many deaths as communicable diseases, maternal, perinatal and nutritional conditions combined in LMICs [7].

Impact of NCDs on individual, societal and national economic development is higher in LMICs due to coexistence with an equally high burden of communicable diseases, reproductive and child health challenges as well as violence and injuries within fragile health systems [8,9]. These countries have poor capacity for diagnosis of NCDs, large-scale public health interventions and management of complications arising from poor treatment or late diagnosis [10].

There are several socio-demographic determinants of the prevalence of NCDs and their risk factors. The major NCD risk factors; tobacco use, harmful use of alcohol, physical inactivity and unhealthy diets, are modifiable behaviors established during adolescence or young adulthood and cause NCDs later in life [11]. High rates of poverty and inequality in the region exacerbate the challenges; living in low-income neighborhoods is an important precursor for NCD risks factors [9]. Even though NCDs cause two out of three deaths in women and the absolute number of NCD deaths in women is similar to men (16.2 versus 18.4 million), less focus is given to NCDs in women globally compared with reproductive and maternal health [1,3].

Determining how NCD prevalence as well as risk factors differ across different gender, wealth quintiles, education level and place of residence can provide public policy and health authorities with invaluable knowledge on designing and implementing intervention packages and policies to address the NCD pandemic. The survey was carried out in Kenya for the first time in 2015, with collection of data on behavioural risk factors, medical history, physical measurements and biochemical tests. Understanding NCD dynamics across socio-economic strata is vital for planning control programmes. This secondary analysis focused on the relationship between age, gender, residence and socio-economic strata and the prevalence of various NCDs and their risk factors, from the survey findings.

## Methods

**Overview of STEPS Kenya, 2015:** the WHO STEPwise survey is a tool developed by the World Health Organization (WHO) to enable countries to systematically undertake surveillance on NCD risk factors worldwide, with tools and methods that are easily reproducible and enable comparison of results from different countries [12].

**Study type, site and population:** data for this study were obtained from the 2015 Kenya STEPs survey. The Kenya STEPwise survey was a cross-sectional household survey targeting adults between the ages of 18 and 69 years conducted between April and June 2015 utilizing the WHO STEPwise tool which is a cross-culturally validated survey tool used to assess burden of leading non-communicable diseases and their associated lifestyle risk factors in a nationally representative sample. The focus of the survey was the four main behavioural risk factors of NCDs (tobacco use, harmful use of alcohol, unhealthy diets and physical inactivity), the four key biological risk factors for NCDs (overweight and obesity, raised blood pressure, raised blood lipids and raised blood glucose) as well as awareness for cervical cancer, burden of unintentional injuries and oral health.

**Sampling:** this survey involved a three-stage cluster sample design involving selection of clusters from the national sample surveys and evaluation programme (NASSEP V) sampling frame from the Kenya National Bureau of Statistics, developed using the enumeration areas generated from the 2009 Kenya population and housing census. Two-hundred clusters (100 urban and 100 rural) were selected with a uniform sample of 30 households being sampled from each cluster in the second stage and an eligible individual respondent sampled using the Kish grid method of sampling in the third stage.

A total of 6000 households were sampled targeting one individual randomly selected from all eligible household members using the Kish method. Of the 4754 participants who gave consent, a total of 4500 individuals were successfully interviewed giving a response rate of 75%. To produce unbiased estimates, sampling weights were calculated as the inverse or reciprocal of all the selection probabilities at all the stages mentioned above. Further, the weights were adjusted to cover individual non-responses. Post stratification adjustments were done to align with the population projections according to age-sex categories.

**Data collection and definitions:** the survey entailed a sequential process consisting of three “steps” of information gathering as follows: step 1 entailed interviews on demographic information and selected major health risk behaviors; step 2 involved anthropometric measurements of height, weight, waist and hip circumference as well as blood pressure and heart rate; step 3 involved biochemical measurements of fasting blood glucose, triglycerides and cholesterol levels. A detailed description of the approach, process and measurements for the Kenya STEPwise survey is available elsewhere [13].

Central obesity was defined as waist-hip ratio of more than 0.85 in men and 0.9 in women. Type II diabetes was defined as fasting blood sugar of 7 mmol/l and above for the first time during the survey, while those with sugars of between 6.0 and 6.9 mmol/l were labeled as prediabetes. Hypertension was defined as systolic blood pressure ≥140 mm Hg and/or diastolic blood pressure ≥90 mm Hg. Heavy episodic drinking was defined as six or more standard drinks in a single drinking occasion. Current tobacco use was defined as use of any tobacco products in the past 30 days. Unhealthy diet was defined as intake of less than five servings of fruits and vegetables in a day, addition of salt at the table while eating or intake of processed foods high in salt or addition of sugar to drinks already served with sugar or intake of processed foods or drinks high in sugar, daily. Physical inactivity was defined as obtaining less than 150 min of moderate intensity physical activity throughout the week or less than 75 min of vigorous intensity physical activity throughout the week or less than an equivalent combination of moderate and vigorous intensity derived from WHO definition of physical activity [14]. The WHO risk estimation model for cardiovascular disease (CVD) risk where age, sex, smoking, blood pressure, blood cholesterol and presence of diabetes were used as basis for estimating the 10-year CVD risk that represented a risk of a fatal or non-fatal cardiovascular event (meaning death from or developing cardiovascular disease) A risk at or above 30 percent is considered high and cost-effective to treat.

**Data analysis:** we conducted a further analysis of the STEPS dataset for the current study. The outcome variables for this study included prevalence of hypertension, heavy episodic drinking, smoking, central obesity, non-use of seatbelts while driving in a vehicle or not wearing helmets while riding a bicycle or motorcycle.

The outcome variables were then compared across the various independent variables used in this study including sex, age, marital status, level of education, occupation, residence and socio-economic status (wealth band). Logistic regression analyses were used to compute adjusted odds ratios (aOR) for each exposure variable while controlling for all the other variables (confounders) in the model. Using the “svy” method in STATA, we created estimates that adjust for the complex, multi-level sampling design, including stratifying by Kenyan regions and enumeratotion areas. We computed adjusted odds ratios for each exposure variable while controlling for all the other variablesin the model with 95% confidence intervals that excluded the null (AOR equal to 1.0) considered statistically significant. This secondary analysis included 4484 respondents after omitting records that had missing values for the independent and dependent variables from the initial survey sample size of 4500 respondents.

## Results

**Overview of the findings:** of the 4484 individuals included in this secondary analysis, 60% were female, 42% had secondary education and above while 13% had no formal education. The mean age was 40.5 years (95%CI: 39.9-41.1 years). Sixty-six percent of the respondents were married, 40% were unemployed while 62% were rural residents (Table 1). Prevalence and correlates of selected NCD risk factors across socio-demographic strata. NCD risk factors distribution varied across socio-demographic strata.

**Table 1:** socio-demographic characteristics of the survey population, Kenya STEPS, 2015

Characteristic	Weighted, n	% proportions (95% CI)
**Gender**		
Male	1791	39.9 (38.3, 42.0)
Female	2693	60.1 (57.8, 61.9)
**Age groups**		
18-29	2062	46.0 (43.6, 48.4)
30-39	1045	23.3 (21.6, 25.1)
40-49	695	15.5 (14.1, 17.0)
50-59	443	9.9 (8.8, 11.0)
60-69	239	5.3 (4.7, 6.1)
**Marital status**		
Not married	1039	23.2 (21, 25.5)
Married	2938	65.5 (63.1, 67.8)
Formerly married	507	11.3 (10, 12.7)
**Residence**		
Rural	2776	61.9 (59.4, 64.4)
Urban	1708	38.1 (35.6, 40.6)
**Education level**		
<12 years of formal education	2606	58.1 (55.7, 60.4)
≥12 years of formal education	1877	41.9 (39.5, 44.3)
**Wealth band**		
Poorest	848	18.9 (17.4, 20.5)
Second	937	20.9 (19.3, 22.6)
Middle	818	18.3 (16.8, 19.8)
Fourth	832	18.6 (16.7, 20.6)
Richest	1049	23.4 (21.0, 25.9)
**Occupation**		
Unemployed	1799	40.1 (37.9, 42.4)
Employed	2685	59.9 (57.6, 62.1)

CI: confidence interval

**Current tobacco use:** compared to females, males had a higher prevalence of current tobacco use (19.9% versus 0.9%, p<0.001). Respondents with less than 12 years of formal education had higher prevalence of tobacco use (12.1 versus 7.6%, p=0.044). Employed respondents had higher prevalence of tobacco use (12.4% versus 6.9%, p=0.021). Tobacco use increased with age (Table 2). Males had eight times higher odds of tobacco use (aOR 7.63, 95% CI 5.63, 10.33) compared with females. Rural dwellers had 35% less odds of tobacco use (aOR 0.65, 95% CI 0.49, 0.86) compared with their urban counterparts. Respondents with at least 12 years of formal education had 89% less odds of tobacco use (aOR 0.11, 95% CI 0.07, 0.17) compared with those with less education (Table 3).

**Table 2:** prevalence of various NCD risk factors across demographic and socio-economic strata, Kenya STEPS, 2015

	Prevalence (%)								
Variable	n	Current tobacco use	Harmful alcohol use	Hypertension	Non-use of helmets	Non-use of safety belts	Central obesity	Type 2 diabetes	Multiple/≥4 NCD risk factors
**Gender**									
Male	1791	19.9	18.1	24.2	89.5	79.8	32.9	2.0	88.4
Female	2693	0.9	2.2	25.0	56.0	56.0	49.5	2.8	82.6
**Education level**									
<12 years of formal education	2606	12.1	6.3	27.3	81.7	73.9	51.5	1.4	85.4
≥12 years of formal education	1877	7.6	2.6	23.5	54.1	53.9	30.8	1.3	84.6
**Residence**									
Rural	2776	9.2	4.6	24.7	75.6	64.3	43.4	1.9	85.9
Urban	1708	11.8	5.0	24.9	69.0	67.4	42.0	3.4	85.0
**Occupation**									
Unemployed	1799	6.9	9.3	24.1	74.8	63.2	46.3	0.7	84.2
Employed	2685	12.4	1.6	25.9	72.1	67.1	40.6	1.8	86.5
**Wealth band**									
Poorest	848	8.9	5.9	19.4	76.1	61.4	45.5	1.6	82.2
Second	937	10.4	5.3	24.3	76.9	58.8	40.6	1.5	86.1
Middle	818	11.6	5.1	27.5	78.1	66.4	44.6	2.0	86.6
Fourth	832	12.1	4.4	24.7	66.8	69.6	39.7	3.0	89.3
Richest	1049	8.4	3.3	29.0	68.6	71.0	43.9	5.2	83.7
**Age group**									
18-29	2062	7.4	4.0	23.9	48.2	27.5	13.5	0.6	81.3
30-39	1045	12.6	12.0	29.3	83.7	67.6	37.3	2.3	87.5
40-49	695	12.8	12.9	21.5	97.1	92.8	62.2	5.8	91.4
50-59	443	11.6	10.4	25.7	80.8	69.8	68.2	7.4	89.4
60-69	239	13.7	16.7	31.8	85.1	56.1	70.3	6.0	90.7

**Table 3:** socio-demographic determinants of the main modifiable NCD risk factors, Kenya STEPS, 2015

Predictor	Physical inactivity		Current tobacco use		Heavy episodic alcohol use		Unhealthy diets	
	aOR* (95% CI)	P-value	aOR*(95% CI)	P-value	aOR* (95% CI)	P-value	aOR* (95% CI)	P-value
**Sex**								
Female	1.00 (ref)		1.00 (ref)		1.00 (ref)		1.00 (ref)	
Male	1.15 (0.86,1.53)	0.338	7.63 (5.63, 10.33)	<0.001	9.9 (5.3,18.8)	<0.001	1.33 (1.04, 1.70)	0.24
**Age group**								
18-29	1.00 (ref)		1.00 (ref)		1.00 (ref)		1.00 (ref)	
30-39	1.01 (0.69,1.47)	0.964	1.76 (1.14, 2.74)	0.011	1.7 (1.1,2.7)	0.05	0.83 (0.70, 0.98)	0.030
40-49	0.54 (0.35,0.83)	0.005	1.13 (0.74, 1.72)	0.585	1.9 (1.0,3.5)	0.05	0.68 (0.56, 0.83)	<0.001
50-59	0.63 (0.47,0.89)	0.067	1.33 (0.91, 1.94)	0.137	1.2 (0.8,1.8)	0.46	0.53 (0.42, 0.67)	<0.001
60-69	0.59 (0.37,0.94)	0.027	0.81 (0.45, 1.46)	0.481	1.7 (1.0,3.0)	0.07	0.46 (0.33, 0.62)	<0.001
**Residence**								
Urban	1.00 (ref)		1.00 (ref)		1.00 (ref)		1.00 (ref)	
Rural	0.66 (0.47,0.92)	0.014	0.65 (0.49, 0.86)	0.003	0.6 (0.4,1.0)	0.04	1.19 (0.89, 1.59)	0.239
**Years in formal schooling**								
<12 years	1.00 (ref)		1.00 (ref)		1.00 (ref)		1.00 (ref)	
≥12 years	0.46 (0.29,0.73)	0.001	0.11 (0.07, 0.17)	<0.001	1.5 (0.8,2.8)	0.21	1.21 (0.95, 1.55)	0.120
**Wealth band**								
Poorest	1.00 (ref)		1.00 (ref)		1.00 (ref)		1.00 (ref)	
Second	0.88 (0.54,1.42)	0.587	0.68 (0.45, 1.04)	0.072	0.8 (0.4,1.6)	0.45	0.8 (0.4,1.6)	0.45
Middle	1.16 (0.71,1.91)	0.549	0.58 (0.37, 0.91)	0.019	0.7 (0.4,1.5)	0.38	1.07 (0.78, 1.47)	0.38
Fourth	1.63 (0.99,2.67)	0.054	0.61 (0.38, 0.97)	0.037	0.8 (0.4,1.8)	0.64	1.75 (1.38, 2.21)	<0.001
Richest	2.42 (1.42,4.13)	0.001	0.63 (0.38, 1.06)	0.082	1.7 (0.8,3.8)	0.18	1.64 (1.26, 2.14)	<0.001
**Employment status**								
Unemployed	1.00 (ref)		1.00 (ref)		1.00 (ref)		1.00 (ref)	
Employed	1.32 (0.75, 2.34)	0.334	0.67 (0.52, 0.85)	0.001	0.58 (0.45, 0.76)	<0.001	0.84 (0.58, 1.21)	0.355

* aOR: adjusted odds ratio; ref: reference; CI: confidence interval

**Harmful use of alcohol:** males had a higher prevalence of harmful alcohol use (18.1% versus 2.2%, p<0.001). Respondents with less than 12 years of formal education had higher prevalence of harmful alcohol use (6.3% versus 2.6%, p=0.019). Unemployed respondents had higher prevalence of harmful use of alcohol compared with those in employment (9.3% versus 1.6%, p=0.028). Harmful use of alcohol also increased with age (Table 2). Males had 10 times higher odds of harmful use of alcohol (aOR 9.9, 95% CI 5.3, 18.8) compared with females. Those in employment had 42% less odds of harmful alcohol use (aOR 0.58, 95% CI 0.45, 0.76) compared with the unemployed.

**Non-use of safety equipment (helmets and safety belts):** males had a higher prevalence of non-use of helmets (89.5% versus 56.0%, p=0.031) and seat belts (79.8 versus 56.0%, p=0.027) compared with females. Respondents with less than 12 years of formal education had higher prevalence of non-use of helmets (81.7% versus 54.1%, p=0.03) and seat belts (73.0% versus 53.9%, p=0.039) compared with those who had spent at least 12 years in school. Non-use of helmets and safety belts increased with age (Table 2).

**Central obesity:** females had higher prevalence of central obesity (49.5% versus 32.9%, p=0.017) compared with males. We found no difference in prevalence of central obesity between rural and urban residents (43.4% versus 42.0%, p=0.780). There were no differences in central obesity according to the wealth status, but it increased with age (Table 2).

**Type 2 diabetes:** respondents in the highest wealth quintile had higher prevalence of type II diabetes compared with those in the lowest (5.2% versus 1.6%, p=0.008). Urban residents had higher prevalence of type 2 diabetes compared with rural residents (3.4% versus 1.9%, p=0.041). Type 2 diabetes prevalence also increased with age (Table 2).

**Hypertension and the 10-year CVD risk from multiple NCD risk factors:** prevalence of hypertension and 10 years CVD risk had no major variation or correlations across socio-economic strata.

**Socio-demographic correlates of the main modifiable NCD risk factors:** several independent associations were identified between selected socio-demographic variables and the main modifiable NCD risk factors. Males had eight times higher odds of tobacco use (aOR 7.63, 95% CI 5.63, 10.33) and 10 times higher odds of harmful use of alcohol (aOR 9.9, 95% CI 5.3, 18.8) compared with females. There were however, no differences in their consumption of unhealthy diets compared to the females. Rural dwellers had 34% less odds of physical inactivity (aOR 0.66, 95% CI 0.47, 0.92) and 35% less odds of tobacco use (aOR 0.65, 95% CI 0.49, 0.86) compared with their urban counterparts. They equally had a 40% less odds of heavy episodic drinking compared to the urban dwellers. Respondents with at least 12 years of formal education had 89% less odds of tobacco use (aOR 0.11, 95% CI 0.07, 0.17). Those in the wealthiest quintile had 64% higher odds of unhealthy diets (aOR 1.64, 95% CI 1.26, 2.14) compared with those in the poorest. They also had a 2.5 times higher risk of sedentary lifestyles with physical inactivity compared with those in the poorest quintile. Those in employment had 42% less odds of harmful alcohol use (aOR 0.58, 95% CI 0.45, 0.76) and 32% less odds of current tobacco use compared with the unemployed (Table 3).

**Figure 1:**
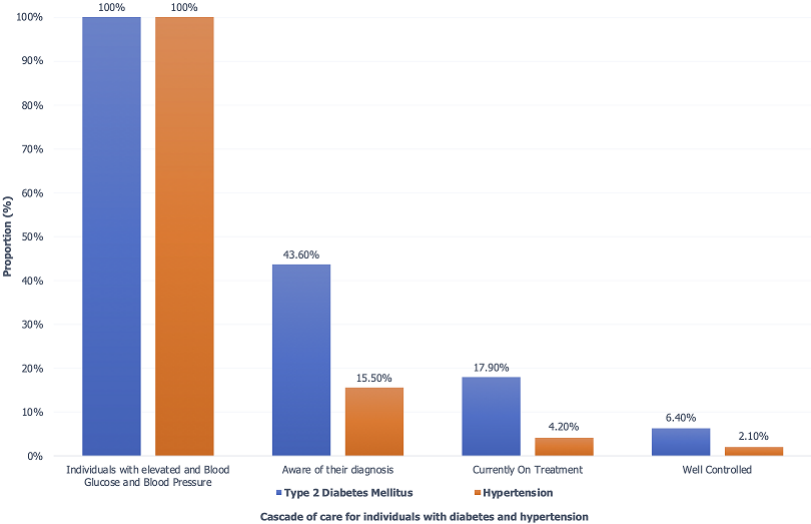
management cascade for type II diabetes mellitus and hypertension in Kenya from awareness to control

**Care cascade for diabetes type II and hypertension:** of the participants with elevated blood pressure, only 16% were aware of their diagnosis, 4% were currently on hypertension treatment and only 2% had well controlled blood pressure under medication. Of the participants with elevated blood glucose, 44% were aware of their diagnosis, only 18% of them were on diabetes treatment and of these, only 6% were well controlled. Medication use varied by age and sex; in those diagnosed with type 2 diabetes, 57% of females and 17% of males were using medications at the time of survey, 53.9% among urban residents and 28.3% among rural residents, those in the age bracket 30-44 years reported the highest current use (67 percent). For diagnosed hypertensive respondents, 24% of women and 18% of men were taking anti-hypertensive medication with only 2.1% of them achieving the desired control (Figure 1).

## Discussion

We noted differences in NCD prevalence and risk across gender, age and place of residence. More males had higher prevalence of harmful alcohol and tobacco use; females had higher prevalence of central obesity. Less educated respondents had higher prevalence of non-use of safety belts and helmets during transportation. Elevated blood glucose was more prevalent in the wealthiest quintile compared with the lowest. Gender, residence, education level and wealth status were independently associated with various NCD risk factors. Awareness, treatment and achievement of control were low for both elevated blood pressure and blood glucose. This analysis focuses on eight out of the nine items identified for follow-up and surveillance by the WHO global action plan for NCDs; reduction in mortality from the main NCDs, reduction in alcohol, tobacco use, unhealthy diets, physical inactivity, diabetes, hypertension and obesity as well as increase availability of treatment [15]. This study identified various socio-demographic attributes of the various NCD risk factors, that are amenable to public health to enable Kenya to attain the stated targets. While the gender, education and rural urban dwelling have been demonstrated as risk factors of NCD in other studies, the marked difference in this analysis warrants their deliberate consideration in preventive and curative policies in an integrated, multi-sectoral and whole of government approach.

Males had higher prevalence of smoking and harmful alcohol use compared to females; this gender difference has been consistent in previous studies and surveys [16,17]. Observed differences in drug and substance use between the genders is as a result of interactions among biological, environmental, sociocultural and developmental influences [18]. While this trend has been observed in various settings, an increase in tobacco use among female youths in LMICs has been noted [19]. The gender difference in tobacco use is less among the youth than in adults; the global youth tobacco survey Kenya 2013 reported tobacco use prevalence of 12.8% for boys and 6.7% for girls [20].

Central obesity has been shown to be a better marker of cardiovascular and other NCD risk compared to body mass index [21]. In this study, females and older individuals had higher prevalence of central obesity. A study in Saudi Arabia also identified sex and age as important factors associated with obesity [22]; the global burden of disease prevalence of overweight and obesity study in 2013 found a lower difference, 37.0% in men and 38.0% in women [23]. We found no difference in central obesity prevalence across residence or wealth strata. This may be as a result of urbanization of rural areas and the expansion of the peri-urban territories, eliminating the expected difference.

Non-use of protective gear during transportation differed by level of education; more educated respondents were more likely to use helmets or seat belts when travelling compared to those with less education. This may be due to low-risk perception and safety awareness as well as higher prevalence of harmful alcohol use among those with less education [24]. Sex, education level, residence and wealth status were noted as the main determinants of current tobacco use, harmful use of alcohol, low physical activity and unhealthy diets. Male sex was associated with tobacco and harmful alcohol use, higher wealth was associated with unhealthy diets and being more educated was negatively associated with alcohol and tobacco use. Males are more prone to risky lifestyles; this may be partly reduced through education. Wealthier residents, especially in urban areas, tend to adopt unhealthy eating habits, as a lifestyle change as well as availability of unhealthy foods. These associations have been observed in other studies as well [25-27].

Majority of the respondents with hypertension or type 2 diabetes were not aware of their diagnosis and even among those aware, most were neither on treatment nor controlled for those already on treatment. In addition to low-risk perception by the patients regarding the complications arising from these two conditions [28], this may also be a result of the health care system that is not optimized to tackle non communicable diseases especially at the primary care level. Integration of NCD care at the primary care level may foster better awareness, uptake of early diagnosis and compliance to treatment especially for the men. There was noted a higher proportion of women on treatment for hypertension and diabetes. This may reflect a poor health seeking behavior of Kenyan men as shown in other studies of seeking health among men and women and may explain the higher proportion of men with long term complications of diabetes like amputations compared to women in the tertiary hospitals in Kenya.

**Limitations and strengths:** self-reporting for some variables, for instance use of safety belts and helmets, alcohol intake, smoking and dietary history were liable to information and recall bias. However, several outcome variables were measured during the survey, including fasting blood sugar, blood pressure and waits-hip circumference. To improve recall, food cards with portion sizes estimation were used during administration of the food frequency questionnaire while picture sample alcoholic beverages were used to estimate alcohol consumption. Due to the limitation of the survey tools, it was not possible to identify the individual factors behind some of our findings, for instance, the reasons for non-use of medications for diagnosed diabetic or hypertensive patients. Finally, exclusion of participants with missing data could have prevented us from identifying important differences in NCD risk factor prevalence in the Kenyan population.

This being a dataset from a nationally representative survey on NCD risk factors, the findings may be generalized to the entire Kenyan population and considering that the analysis focused on all the main NCD risk factors identified by WHO in the global action plan for NCDs; these findings are vital in policy evaluation and formulation within Kenya. They are also comparable to similar WHO member states who have deployed the STEPS questionnaire. By identifying the socio-demographic determinants of the NCD risk factors, this analysis offers opportunities for ‘quick wins´ in the public health response to NCDs in Kenya.

## Conclusion

Socio-demographic factors are important determinants of NCD and risk factor prevalence in Kenya. Addressing these determinants requires a multisectoral, whole-of-government and whole-of-society approach as some enablers of behavior change are in jurisdictions beyond the control of the ministry of health. An emphasis on universal basic education access (up to secondary level) is desirable to reduce alcohol and tobacco use as well as improve road safety. Targeted advocacy on physical inactivity and unhealthy diets especially to the urban dwellers and wealthier individuals is required to foster behavior change and adoption of healthier lifestyles. Equally, improvement of the built environment in urban settings to enhance physical activity as well as the creation of open spaces with improved security and access should be considered to improve uptake of physical activity especially in urban settings. Safe and accessible walkways may also reduce the use of motorized transport in urban settings thus improving the uptake of physical activity.

As part of improving treatment outcomes, opportunities for early detection and linkages to treatment and follow-up for hypertension and diabetes, especially among men is required. Integration of NCDs into existing public health programs to take advantage of synergies and broaden opportunities of awareness creation, diagnosis and linkage to care is also essential. Access to information and care especially at the primary care level would go a long way to halt and reverse the burden of non-communicable diseases. Information may need to be customized to the understanding of the individuals and delivered in different platforms to facilitate behavior change. Workplace wellness program focusing on awareness, early detection, treatment linkages and compliance can improve NCD outcomes.

### What is known about this topic

- Gender and socio-economic differences influence the prevalence of NCDs and their risk factors;
- Low levels of education is associated with higher prevalence and risk factors of NCD like tobacco use, harmful alcohol use, non-use of helmets and seat belts.

### What this study adds

- Living in low-income neighborhoods is an important precursor for NCD risks factors. High rates of poverty and inequality exacerbate this challenge;
- The majority of individuals living with hypertension or type 2 diabetes were not aware of their diagnosis and even among those aware, most were neither on treatment nor well controlled;
- Medication use and compliance for those diagnosed with type 2 diabetes and hypertension varies by age, gender and locality with rural dwellers being at a higher risk of not being on treatment for elevated blood glucose and hypertension indicating geographical or health system related barrier to access for NCDs care.

## References

[1] Murray CJ, Vos T, Lozano R, Naghavi M, Flaxman AD, Michaud C (2012). Disability-adjusted life years (DALYs) for 291 diseases and injuries in 21 regions, 1990-2010: a systematic analysis for the global burden of disease study 2010. Lancet.

[2] Hosseinpoor AR, Bergen N, Kunst A, Harper S, Guthold R, Rekve D (2012). Socioeconomic inequalities in risk factors for non communicable diseases in low-income and middle-income countries: results from the World Health Survey. BMC Public Health.

[3] Salomon JA, Wang H, Freeman MK, Vos T, Flaxman AD, Lopez AD (2012). Healthy life expectancy for 187 countries, 1990-2010: a systematic analysis for the global burden disease study 2010. Lancet.

[4] Marquez PV, Farrington JL (2013). The challenge of non-communicable diseases and road traffic injuries in sub-Saharan Africa: an overview.

[5] Lozano R, Naghavi M, Foreman K, Lim S, Shibuya K, Aboyans V (2012). Global and regional mortality from 235 causes of death for 20 age groups in 1990 and 2010: a systematic analysis for the global burden of disease study 2010. Lancet.

[6] World Health Organization (2011). Global status report on noncommunicable diseases 2010.

[7] Miranda JJ, Kinra S, Casas JP, Davey Smith G, Ebrahim S (2008). Non-communicable diseases in low-and middle-income countries: context, determinants and health policy. Trop Med Int Healt.

[8] Bollyky TJ, Templin T, Cohen M, Dieleman JL (2017). Lower-income countries that face the most rapid shift in noncommunicable disease burden are also the least prepared. Health Aff (Millwood).

[9] Bloom DE, Cafiero E, Jané-Llopis E, Abrahams-Gessel S, Bloom LR, Fathima S (2012). The global economic burden of noncommunicable diseases. PGDA Work Pap.

[10] McQueen DV (2013). Global handbook on noncommunicable diseases and health promotion. Springer Science & Business Media.

[11] Naik R, Kaneda T (2015). Noncommunicable diseases in africa: youth are key to curbing the epidemic and achieving sustainable development. Population Reference Bureau.

[12] World Health Organization (2005). WHO STEPS surveillance manual: the WHO STEPwise approach to chronic disease risk factor surveillance.

[13] Ministry of Health (2015). Kenya STEPwise survey for non communicable diseases risk factors 2015 report.

[14] World Health Organization (2002). Diet. physical activity and health report of the fifty-fifth World Health Assembly. WHO.

[15] World Health Organization (2013). Global action plan for the prevention and control of noncommunicable diseases 2013-2020. WHO Geneva.

[16] World Health Organization (2014). Kenya-global adult tobacco survey 2014.

[17] World Health Organization (2014). Global status report on alcohol and health 2014.

[18] Becker JB, McClellan ML, Reed BG (2017). Sex differences, gender and addiction. J Neurosci Res.

[19] Drope J, Schluger N, Cahn Z, Drope J, Hamill S, Islami F (2018). The tobacco atlas.

[20] International Institute for Legislative Affairs Kenya (2013). Global youth tobacco survey (GYTS, 2013) Kenya: factsheet.

[21] Savva SC, Lamnisos D, Kafatos AG (2013). Predicting cardiometabolic risk: waist-to-height ratio or BMI: a meta-analysis. Diabetes Metab Syndr Obes.

[22] Memish ZA, El Bcheraoui C, Tuffaha M, Robinson M, Daoud F, Jaber S (2014). Obesity and associated factors-Kingdom of Saudi Arabia, 2013. Prev Chronic Dis.

[23] Ng M, Fleming T, Robinson M, Thomson B, Graetz N, Margono C (2014). Global, regional and national prevalence of overweight and obesity in children and adults during 1980-2013: a systematic analysis for the global burden of disease study 2013. Lancet.

[24] Sami A, Moafian G, Najafi A, Aghabeigi MR, Yamini N, Heydari ST (2013). Educational level and age as contributing factors to road traffic accidents. Chinese J Traumatol.

[25] Wang Q, Shen JJ, Sotero M, Li CA, Hou Z (2018). Income, occupation and education: are they related to smoking behaviors in China, Schooling CM, ed. PLoS One.

[26] Ssewanyana D, Abubakar A, van Baar A, Mwangala PN, Newton CR (2018). Perspectives on underlying factors for unhealthy diet and sedentary lifestyle of adolescents at a Kenyan coastal setting. Front Public Heal.

[27] He Z, Bishwajit G, Yaya S (2018). Prevalence of alcohol and tobacco use among men and women in Namibia. Int J Environ Res Public Health.

[28] Kagaruki GB, Mayige MT, Ngadaya ES, Kilale AM, Kahwa A, Shao AF (2018). Knowledge and perception on type2 diabetes and hypertension among HIV clients utilizing care and treatment services: a cross sectional study from Mbeya and Dar es Salaam regions in Tanzania. BMC Public Health.

